# Supplementary material for: Magnitude and antimicrobial susceptibility profiles of Gram-Negative bacterial isolates among patients suspected of urinary tract infections in Arba Minch General Hospital, southern Ethiopia
Source: PLoS One. 2022 Dec 30;17(12):e0279887. doi: 10.1371/journal.pone.0279887 (PMC9803306; doi:10.1371/journal.pone.0279887)
Supplement: Attachment — Submitted filename: Reviewers comment.docx [file pone.0279887.s002.docx]

**Title:** Carbapenemase Producing Gram Negative bacterial pathogens and Associated Factors among Patients Suspected of Urinary Tract Infections Attending Arba Minch General Hospital, southern Ethiopia

I would like to appreciate the Editors of PLOS One invitation to contribute to reviewing process of this interesting study.

**Comments**

This article This study determined the prevalence of carbapenemase producing Gram-negative uropathogens and associated factors among patients suspected of urinary tract infections. however, there are multiple issues with this manuscript.

**Introduction of the study:** Failed to provide adequate background for the study. the rationale of the study and the research problem not well stated. There are incomplete and lack of logical flow of the sentences and paragraphs. Also, the scientific literature not well cited (e. g from page 78 to 86).

**Methodology:** The authors used a routine investigation of carbapenemase production. It would be better to perform molecular characterization of carbapenemase genes of blaOXA-48 like, bla NDM-1 and other common genes, which are now become predominant determinants to cause carbapenem resistant in Gram-negative pathogens worldwide.

**Results, Discussions and Abstract:** to be modified accordingly.

**Others**

The writing style needs to be modified.
